# Complete chloroplast genome of *Prunus canescens*: an endemic shrub in China

**DOI:** 10.1080/23802359.2019.1624641

**Published:** 2019-07-16

**Authors:** Xin Chen, Xiaojuan Zong, Qingzhong Liu, Boqiang Tong, Li Xu, Lisi Zhang, Po Hong

**Affiliations:** a Shandong Provincial Key Laboratory of Fruit Tree Biotechnology Breeding, Shandong Institute of Pomology, Shandong Academy of Agricultural Sciences, Taian, Shandong, P. R. China;; b Shandong Forest Germplasm Resources Center, Jinan, Shandong, P. R. China

**Keywords:** *Prunus canescens*, chloroplast genome, Illumina sequencing

## Abstract

*Prunus canescens* is an endemic cherry species in China, which is distributed in Shaanxi, Gansu, Hubei, and Sichuan provinces of China. The chloroplast (cp) genome of *P. canescens* is 157,890 bp in size containing 125 unique genes, including 8 rRNA genes, 37 tRNA genes, and 80 protein-coding genes (PCGs). Phylogenetic analysis exhibited that *P. canescens* is most related to *P. pseudocerasus*.

*Prunus canescens*, an endemic species in China, is a species of cherry of the family Rosaceae. It is a deciduous shrubby tree and reaches a height of 2 m. It is distributed in Shaanxi, Gansu, Hubei, and Sichuan province of China (Instituto Botanico Boreali-Occidentali Academiae Sinicae 1974). The altitude of its natural mountain habitat is between 1300 and 1600 m. *Prunus canescens* is a parent of ‘GiSeLa’, which is one of the most popular rootstocks all over the world (Whiting et al. 2005). Hence, the genomic sequence information is urgently needed to promote molecular evolution, systematics research, conservation, and utilization of *P. canescens*. The objectives of the present study were to reconstruct the cp genome of *P. canescens* and assess phylogenetic relationships among different species within the family Rosaceae.

Leaves were sampled from a mature *P. canescens* tree at Maiji Mountain, Maiji, Tianshui, Gansu, China (34°21′7.91″N, 106°0′14.89″E) and chilled with liquid nitrogen immediately. The voucher specimen (accession no. TS_2019_Maiji_Taian) was stored at –80 °C in Shandong Institute of Pomology (SDIP). Genomic DNA (gDNA) was obtained from homogenized leaf tissues using a modified CTAB protocol (Doyle and Doyle 1987). The quantity and quality of the purified gDNA were detected by Nanodrop 8000 and via the Agilent 2100 Bioanalyzer. A library with 350 bp fragments inserted was constructed with 1 μg purified DNA and high-throughput sequenced with paired end (PE) reads of 2 × 150 bp on Illumina Hiseq 2500 platform. Raw reads were filtered and trimmed to remove low quality and contaminated reads by trim_galore v0.4.4. Totally 7.9 Gb of clean data were aligned to the *Prunus cerasoides* complete cp genome (GenBank no. NC_035891) (Xu et al. 2018) as a reference using bowtie2 v2.2.4 (Langmead and Salzberg 2012) and assembled with SPAdes v3.10.1 (Bankevich et al. 2012). The final cp genome was annotated using DOGMA (Boore et al. 2004), HMMER 3.1b2 (Finn et al. 2011), and ARAGORN v1.2.38 (Laslett and Canback 2004).

The cp genome of *P. canescens* (GenBank no. MK816299) is 157,890 bp in size with total AT content of 63.3%. It contains a 19,147 bp small and 85,909 bp large single-copy regions with AT contents 65.4 and 69.9%, respectively, and two 26,417 bp inverted repeat regions with AT content 57.5%. In the cp genome of *P. canescens*, there are 125 unique genes, including 8 rRNA genes, 37 tRNA genes, and 80 PCGs. Thirteen genes, including seven PCGs (rps19, rpl23, rpl2, psbA, ndhC, ndhI and atpA), harbour one intron each, while two protein-coding genes (psaA and rpl20) harbour two introns each.

To perform the molecular phylogenetic analysis, 15 published complete cp genomes were aligned by MAFFT v7.307 (Katoh and Standley 2013). Finally, a maximum likelihood (ML) tree was constructed using RAxML v.7.2.6 with the GTRGAMMA model (Stamatakis 2006). The ML phylogenetic tree shows that *P. canescens* is most related to *P. pseudocerasus* (Figure 1).

**Figure 1.**
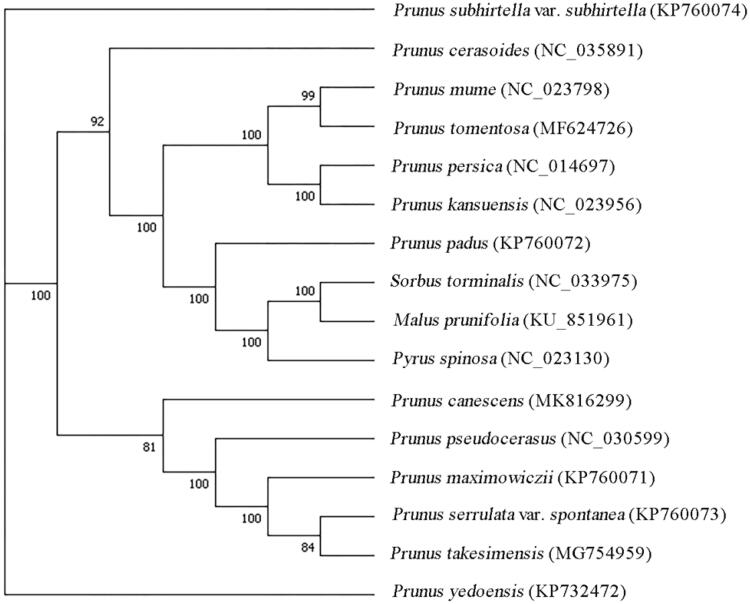
Phylogenetic tree based on 16 complete cp genome sequences. The bootstrap support values are shown next to the branches.
